# Serodiagnosis of Visceral Leishmaniasis in Northeastern Italy: Evaluation of Seven Serological Tests

**DOI:** 10.3390/microorganisms8121847

**Published:** 2020-11-24

**Authors:** Margherita Ortalli, Daniele Lorrai, Paolo Gaibani, Giada Rossini, Caterina Vocale, Maria Carla Re, Stefania Varani

**Affiliations:** 1 Unit of Microbiology, Regional Reference Center for Microbiological Emergencies (CRREM), St. Orsola Malpighi Hospital, University of Bologna, 40100 Bologna, Italy; margherita.ortalli2@unibo.it (M.O.); paolo.gaibani@unibo.it (P.G.); giada.rossini@unibo.it (G.R.); cate.vocale@gmail.com (C.V.); mariacarla.re@unibo.it (M.C.R.); 2 Department of Experimental, Diagnostic and Specialty Medicine, University of Bologna, 40100 Bologna, Italy; daniele.lorrai@studio.unibo.it

**Keywords:** visceral leishmaniasis, serodiagnosis, screening tests, *Leishmania infantum*, rk39 immunochromatographic test

## Abstract

This study compares the performance of seven assays, including two ELISA (Leishmania ELISA IgG + IgM, Vircell Microbiologists; Leishmania infantum IgG ELISA, NovaTec), three rK39-based immunochromatographic tests (rK39-ICTs) (Leishmania Dipstick Rapydtest, Apacor; On Site Leishmania IgG/IgM Combo Rapid Test, CTK Biotech; LEISHMANIA Strip quick Test, Cypress Diagnostic), one indirect immunofluorescent antibody test (IFAT) (Leishmania-Spot IF, BioMérieux), and one western blot (WB) (Leishmania WESTERN BLOT IgG, LDBio Diagnostics) for serodiagnosis of visceral leishmaniasis (VL). Serum samples from 27 VL patients living in northeastern Italy were analyzed, as well as the serum samples from 50 individuals in whom VL diagnosis was excluded. The WB and the IFAT had 96% sensitivity, followed by the ELISA (63% and 74%, respectively). The rK39-ICT exhibited the worst performance among the serological tests, with sensitivities ranging from 52% to 70%. By combining selected ELISA/ICT, the sensitivity of VL detection reached 89%. IFAT and WB outperformed ELISA and rK39-ICT by possessing optimal sensitivity, but their high cost and complexity of execution would not allow their employment as screening tests. In conclusion, the combination of easy-to-perform tests, such as ICT and ELISA, could improve sensitivity in the serodiagnosis of Mediterranean VL.

## 1. Introduction

Visceral leishmaniasis (VL) is a severe infection caused by the sandfly-transmitted protozoa of the *Leishmania donovani* complex [1]. *Leishmania infantum* causes VL in Europe, where the disease is hypoendemic in nine countries; nearly 75% of the cases in the World Health Organization (WHO) European region occur in Albania, Georgia, Italy, and Spain [2]. Several VL outbreaks have occurred in the WHO European region since 2009, including in Madrid in Spain [3], in Bologna and Modena in northeastern Italy [4,5], and in Tbilisi in Georgia [6].

The diagnosis of VL is difficult; efforts have been made to replace the microscopic identification of parasites in bone marrow specimens with more sensitive molecular methods [7,8]. However, despite the amount of evidence regarding the optimal performance of PCR assays, such techniques are restricted to well-equipped laboratories, and standardization of protocols is lacking [9].

Besides parasite detection, serological tools provide good diagnostic accuracy [9,10]. Different serological tests are currently available and widely used for diagnosis, including the rapid immunochromatographic test based on the recombinant protein K39 (rK39-ICT), the indirect fluorescent antibody test (IFAT), the enzyme-linked immunosorbent assay (ELISA), the western blot (WB), and the direct agglutination test [8,9,10].

Even though VL is widespread in Mediterranean Europe, serological tests have been mainly validated in highly endemic regions, including the Horn of Africa, the Indian subcontinent, and Brazil [11]. The aim of this study was to evaluate and compare the performance of seven serological tests in the diagnosis of autochthonous VL from northeastern Italy, by performing a retrospective analysis of sera collected from VL and non-VL patients.

## 2. Materials and Methods

This study was designed as a retrospective comparative study. We obtained samples from patients with suspected VL that were admitted to healthcare facilities in the Emilia-Romagna region (northeastern Italy). Among samples submitted for VL diagnosis at the Regional Reference Centre for Microbiological Emergencies (CRREM), Unit of Microbiology, St. Orsola-Malpighi University Hospital, Bologna (Italy) between 2013 and 2015, 77 serum samples were selected; VL was confirmed in 27 cases and dismissed in 50 cases. Clinical signs suggestive of VL included prolonged fever, splenomegaly, and loss of weight, while laboratory data included anemia, thrombocytopenia, leukopenia, and/or hypergammaglobulinemia [1]. VL diagnosis was confirmed by PCR analysis; parasitic DNA was amplified in peripheral blood and/or bone marrow aspirates by employing simultaneously two real-time PCR assays as described [12]. The protocol of this study was approved by the Ethics Committee of the St. Orsola-Malpighi University Hospital (prot. n.1049/2016). Index tests included the following seven serological assays: IFAT: Leishmania-Spot IF, BioMérieux (Marcy-l’Étoile, France); rK39-ICT: (i) Leishmania Dipstick Rapydtest, Apacor (Wokingham, England), (ii) On Site Leishmania IgG/IgM Combo Rapid Test, CTK Biotech (Poway, CA, USA), (iii) LEISHMANIA Strip quick Test, Cypress Diagnostics (Hulshout, Belgium); ELISA: (i) Leishmania ELISA IgG + IgM, Vircell Microbiologists (Granada, Spain); (ii) Leishmania infantum IgG ELISA, NovaTec (Dietzenbach, Germany); WB: Leishmania WESTERN BLOT IgG, LDBio Diagnostics (Lyon, France) [13]. All tests were performed according to the manufacturers’ instructions. By means of true positive, true negative, false positive and false negative rates, we computed sensitivity, specificity, and accuracy. Statistical analysis was performed by using SPSS v. 20.0 (IBM Corp., Armonk, NY, USA).

## 3. Results

Seventy-seven serum samples from patients with suspected VL were selected, of which 27 were from patients with confirmed VL and 50 were from VL negative patients. The mean age of patients was 44 years (range: 1 month to 87 years). Fifteen patients were children (range: 1 month to 13 years), 11 patients (14%) were HIV-positive, and one patient was immunocompromised with hematological malignancy.

The overall sensitivity, specificity, and accuracy of the index tests are summarized in Table 1. ELISA showed a better performance than ICT. In addition, IFAT and WB showed the best sensitivity, both with a value of 96%. All tests except WB exhibited high specificity values. By restricting results to immunocompetent patients (*n* = 65), the overall sensitivity of the tests increased significantly; the sensitivity range for rK39-ICT was 59–77%, the sensitivity range for ELISA was 68–82%, while IFAT and WB reached a sensitivity of 100%. No significant difference was observed for specificity values (data not shown).

Moreover, by restricting results to the twelve immunocompromised patients, the overall sensitivity was significantly lower for most tests: ICT, ELISA, and IFAT exhibited sensitivities between 20% and 40%, while WB had a sensitivity of 80% (data not shown). Conversely, the overall specificity was 100%.

Next, we calculated the performance of selected test combinations with the aim of reducing false-negative results (i.e., ensuring that a case with a positive result of any test within the combination was counted as positive). By combining the results of rK39-ICT and ELISA, the sensitivity of serological tests in detecting VL cases ranged from 82% to 89% (Table 2). By restricting results to immunocompetent patients, the average sensitivity of the test combinations increased significantly, in particular the combination of Vircell ELISA/Apacor rK39-ICT and the combination of Vircell ELISA/CTK Biotech rK39-ICT exhibited a sensitivity of 96% (78–99 CI 95%).

## 4. Discussion

Despite the availability of several serological tests and the easy-to-use format of some of them, these techniques alone are not always sufficient to identify all cases of VL. For example, serological assays possess limited usefulness in individuals with a previous history of VL, as these tests cannot discriminate between a case of VL relapse and other diseases [7]. Further, the suboptimal performance of serological tests in immunocompromised patients should be taken into consideration [7,8,9,10].

The performance of rK39-ICT has been evaluated in highly endemic country settings with variable results in different geographic locations [11,14]. In addition, recent evidence from the Mediterranean region indicates 83% sensitivity of rK39-ICT in immunocompetent Spanish patients [15] as well as 85–90% sensitivity in VL samples obtained from France, Morocco, and Tunisia [16].

In contrast to other findings on the serodiagnosis of Mediterranean VL, we observed that ICT based on rK39 were less effective than other tests in detecting VL in Italian patients, showing modest sensitivity. We hypothesize that the lower sensitivity that we found for ICT as compared to studies performed in other Mediterranean countries could be due to the presence of genetic variations of the strains circulating in our region that may reflect the molecular diversity of the rK39 homologous sequences [17,18]. These variations may influence the recognition by specific antibodies in VL patients. Alternatively, the dissimilar findings between our study and other studies may be related to the variable performance of rK39-ICT produced by different companies [19].

In this study, IFAT and WB outperformed ELISA and rK39-ICT by possessing optimal sensitivity, but their high cost and complexity of execution would not allow their employment as screening tests. Nevertheless, by performing disjunctive test combinations, we observed that the performance of the ICT/ELISA combination was superior to that of single tests and reached 89% sensitivity. Further, the sensitivity of the selected ICT/ELISA combination increased to 96% by restricting results to immunocompetent patients.

When restricting results to immunocompromised patients, the sensitivity of all serological tests (except for WB) was very low. Since it is well known that serological diagnosis can be unreliable in individuals with impaired immunity, it is always appropriate to associate a molecular test with the serodiagnosis in this patients’ cohort [20].

All assays except WB were highly specific (>96%). The reason for the lower specificity of WB may reflect the high efficiency of this assay in detecting antibodies. In fact, as patients were selected in an endemic area for leishmaniasis [2], asymptomatic carriers of *Leishmania* were probably included in the VL-negative group; this is consistent with a study performed in northwestern Italy showing 7% *Leishmania*-positive blood donors by WB [21]. Thus, a positive result by WB should be carefully interpreted to discriminate between VL and cryptic *Leishmania* infection.

The small study sample and the retrospective nature of the study are the main limitations of this work, while the employment of multiple serological tests is its strength.

Facing an upsurge and a northward spread of VL cases in Europe [2,22], it is crucial to validate methods for the serodiagnosis of VL in the Mediterranean region, including Italy. Our results can inform microbiologists and clinicians on VL serodiagnosis. The selected ICT/ELISA test combination could be useful for improving the sensitivity of screening tests, while WB and IFAT should be recommended as confirmatory tests in the diagnosis of Mediterranean VL.

## Figures and Tables

**Table 1:** Performance of seven serological tests for visceral leishmaniasis (VL) diagnosis.

TEST	Sensitivity %	Specificity %	PPV	NPV	Accuracy %
Apacor ICT	70 (50–85)	96 (85–99)	90 (68–98)	86 (73–93)	87
CTK Biotech ICT	63 (42–80)	98 (88–100)	94 (71–100)	83 (70–91)	86
Cypress ICT	52 (32–71)	100 (91–100)	100 (73–100)	79 (67–88)	83
Vircell ELISA	74 (53–88)	98 (88–100)	95 (74–100)	88 (75–94)	90
NovaTec ELISA	63 (42–80)	100 (91–100)	100 (77–100)	83 (71–91)	87
IFAT	96 (80–100)	100 (91–100)	100 (84–100)	98 (88–100)	99
WB	96 (80–100)	88 (75–95)	81 (63–92)	98 (87–100)	91

Abbreviations: ELISA, enzyme-linked immunosorbent assay; ICT, immunochromatographic test; IFAT, indirect fluorescent antibody test; NPV, negative predictive value; PPV, positive predictive value; WB, western blot. Data are percentages (95% CI, confidence interval).

**Table 2:** Performance of selected test combinations for VL diagnosis.

Test Combination	Sensitivity %	Specificity %
ELISA Vircell + Apacor ICT	89 (70–97)	94 (82–98)
ELISA Vircell + Cypress ICT	82 (61–93)	98 (88–100)
ELISA Vircell + CTK Biotech ICT	89 (70–97)	96 (85–99)
NovaTec ELISA + Apacor ICT	85 (65–95)	96 (85–99)
NovaTec ELISA+ Cypress ICT	82 (61–93)	100 (91–100)
NovaTec ELISA+ CTK Biotech ICT	82 (61–93)	98 (88–100)

Abbreviations: ELISA, enzyme-linked immunosorbent assay; ICT, immunochromatographic test. Data are percentages (95% CI, confidence interval).
